# Patient‐specific pharmacogenomics demonstrates xCT as predictive therapeutic target in colon cancer with possible implications in tumor connectivity

**DOI:** 10.1002/1878-0261.70129

**Published:** 2025-09-24

**Authors:** Marco Strecker, Keren Zohar, Martin Böttcher, Thomas Wartmann, Henry Freudenstein, Maximilian Doelling, Mihailo Andric, Wenjie Shi, Or Kakhlon, Katrin Hippe, Beatrix Jahnke, Dimitrios Mougiakakos, Franziska Baenke, Daniel Stange, Roland S. Croner, Michal Linial, Ulf D. Kahlert

**Affiliations:** ^1^ Molecular and Experimental Surgery, Clinic for Visceral‐, General, Vascular and Transplantation Surgery, University Medicine Magdeburg Otto‐von Guericke University Magdeburg Magdeburg Germany; ^2^ Department of Biological Chemistry, Faculty of Sciences The Hebrew University of Jerusalem Jerusalem Israel; ^3^ Clinic for Hematology and Oncology, University Medicine Magdeburg Otto‐von Guericke University Magdeburg Magdeburg Germany; ^4^ Department of Neurology, The Agnes Ginges Center for Human Neurogenetics Hadassah‐Hebrew University Medical Center Jerusalem Israel; ^5^ Faculty of Medicine The Hebrew University of Jerusalem Israel; ^6^ Institute for Pathology, University Medicine Magdeburg Otto‐von Guericke University Magdeburg Magdeburg Germany; ^7^ Department of Surgery University Medical Center Dresden Dresden Germany; ^8^ National Center for Tumour Diseases Dresden (NCT/UCC), a partnership between DKFZ, Faculty of Medicine and University Hospital Carl Gustav Carus TUD Dresden University of Technology, and Helmholtz‐Zentrum Dresden – Rossendorf (HZDR) Dresden Germany; ^9^ Research Campus Stimulate University of Magdeburg Magdeburg Germany

**Keywords:** chemotherapy resistance, ferroptosis, patient‐derived organoids, tumor metabolism, xCT

## Abstract

Colorectal cancer (CRC) represents the third leading cause of cancer‐related deaths. Integrating cellular and molecular data from individual patients has become valuable for diagnosis, prognosis, and treatment selection. Here, we present a comparative mRNA‐seq analysis of tissue samples from 32 CRC patients, pairing tumors with adjacent healthy tissues. Differential expression gene (DEG) analysis revealed dysregulated metabolic programs. We focused on the impact of overexpressed SLC7A11 (xCT) and SLC3A2, which compose the cystine/glutamate transporter (Xc‐) system. To assess the oncogenic potential of the Xc‐ system, we analyzed gene perturbations from CRISPR screens across various cell types and used functional assays in five primary patient‐derived organoid models. We identified a previously uncharacterized cell surface protein signature predicting chemotherapy resistance and highlighted the causality and potential of pharmacological blockage of ferroptosis as a promising avenue for cancer therapy. Redox homeostasis, ion/amino acid transporters, and regulators of neuronal survival and differentiation were pathways associated with these co‐dependent genes in patient specimens. This study highlights several potential clinical targets for CRC therapy and promotes the use of patient‐derived organoids *oids* to functionally validate *in silico* predictions.

## Graphical abstract

This study integrates transcriptomic profiling of matched tumor and healthy tissues from 32 colorectal cancer patients with functional validation in patient‐derived organoids, revealing dysregulated metabolic programs driven by overexpressed xCT (SLC7A11) and SLC3A2, identifying an oncogenic cystine/glutamate transporter signature linked to chemotherapy resistance, ferroptosis vulnerability, and novel therapeutic targets for precision oncology.

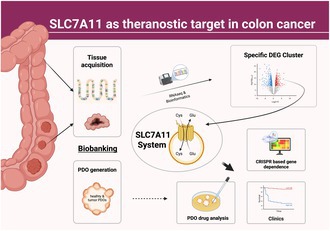

**Abbreviations**
CD44v: CD44 variant
COAD: colorectal adenocarcinoma
CPM: counts per million
CRC: colorectal cancer
DEG: differential expression of gene
DEM: differential gene expression of miRNA
EMT: epithelial–mesenchymal transition
FC: fold change
GO: gene ontology
GPX: glutathione peroxidases
GSH: glutathione
GTEx: genotype‐tissue expression
ICI: immune checkpoint inhibitor
LOF: loss of function
MFI: mean fluorescence intensity
NGS: next‐generation sequencing
OS: overall survival
PCA: principal component analysis
PDO: patient‐derived organoid
PFS: progression‐free survival
READ: rectum adenocarcinoma
RNA‐seq: RNA sequencing
ROS: reactive oxygen species
TCGA: the cancer genome atlas
TMM: trimmed mean of the M‐values
TPM: transcript per million
xCT: cystin/glutamate antiporter

## 1 Introduction

Colorectal cancer (CRC) accounts for 10% of global cancer cases, making it the third most common cancer and the third leading cause of cancer‐related deaths in the United States. Access to extensive cancer patient data enables evaluation of crucial predictive biomarkers needed for optimizing treatment choices. Early diagnosis is crucial for improved survival, currently based on clinical features like age, family history, tumor location, size, and tumor nodes metastasis (TNM) staging [1]. Genetic alterations, particularly recurrent mutations, are also tested for higher precision [2]. While noninvasive tests (*e.g*., blood and stool tests) show promise, their lack of mechanistic or cellular interpretation limits their use [3]. Surgery is the primary treatment for most CRC patients, with chemotherapy being administered for advanced disease [4]. Although the analysis of data from large‐scale, open‐access data platforms from clinical tumor samples has accelerated our knowledge gained on different neoplasms, they often lack proper control datasets or have limitations in the completeness of corresponding clinical metadata, especially post‐surgery clinical follow‐up. Nevertheless, multi‐omics approaches, including diverse cellular and molecular data, have become valuable in influencing CRC diagnosis, prognosis, and treatment selection [5].

Studying tumor cells' metabolic demands, and their capacity to cope with stress, along with characterizing the cancer immune microenvironment, can enhance therapy precision [6, 7]. In any living system, the accessibility of amino acids is crucial for energy production, translation efficiency, and redox homeostasis [8]. It has been shown that cancer cells need large amounts of cysteine and glutathione (GSH) to neutralize the increased intracellular reactive oxygen species (ROS) [9]. Cysteine starvation induces cell death that can be rescued by antioxidants. Most cancer cells rely on the Xc‐heterodimeric amino acid transport system, consisting of SLC7A11 (xCT) and SLC3A2 (heavy chain 4F2hc) [10].

Over the past decade, patient‐derived organoids (PDOs) have become an established three‐dimensional *ex vivo* model that augments traditional cell lines and xenografts in both preclinical pharmacology and individualized therapy development [11]. Generated from small tumor biopsies, PDOs preserve the histological architecture, genomic landscape, and intra‐tumor heterogeneity of their tissue of origin with high fidelity. Crucially, several prospective studies show that drug‐response profiles measured in PDOs mirror clinical outcomes, enabling the prediction of patient‐specific sensitivity or resistance to chemotherapy, targeted agents, and radiotherapy [12].

In this study, we focus on the transcriptional profiles (mRNAs and miRNAs) from 32 Western Caucasian colon cancer patients, each analyzed by comparing their tumor to the healthy tissue. We identified strongly upregulated coding gene sets that signify a mitotic cell signature from colon, cell cycle G2/M checkpoints, and an additional network of dysregulated transporters leading to a metabolic burden. We focused on SLC7A11 and its functional network as an integrator of colon cancer progression. Using functional CRISPR cellular fitness analysis and survival data from large resources, we identified genes carrying clinically relevant properties. An exhaustive bioinformatic analysis explored the potential of Xc‐system status for clinical decisions. While therapy responsiveness and the composition of immune cells did not provide a valid signal for prediction, genes involved in nutrient supply, mitochondria, and redox state were strongly indicative. Further functional investigations using PDOs from patients with CRC showed that therapeutic responses to both standard regimens and xCT inhibitors are significantly modulated by xCT expression levels and the newly identified surface marker profile predicting for therapy response. We illustrate the importance of a multilayer analysis in exposing overlooked cellular processes and targets for improving the clinical and therapeutic management of CRC. Our project highlights the usability of the PDO platform for guiding stratification of therapy resistance levels of tumors as well as supporting early‐stage drug development.

## 2 Methods

### 2.1 RNA‐seq analyses of 32 CRC patients

The mRNA expression levels of all genes (coding and non‐coding) were determined by pairwise analysis of cancerous and healthy tissues obtained from the same patient. Healthy tissue samples were harvested from the aboral margin of the colectomy specimen, at least 5–7 cm distal to the primary tumor, in accordance with national and international guidelines for oncologic resection safety. These normal adjacent tissue samples were macroscopically examined, approved by the pathologist, and underwent extensive washing procedures prior to sequencing to ensure the absence of tumor cell contamination. A total of 32 patients (Table S2) were analyzed with 73 deep sequencing results. Altogether, there were 36 samples marked as tumor (T) and 37 samples marked as healthy (H). Each participant provided at least one sample for T and H. For four participants, the number of samples was higher (Table S1). Each sample was studied by partitioning total RNA by size to support mRNA and miRNA transcriptomics (Table S8).

The study methodologies conformed to the standards set by the Declaration of Helsinki, and all participants confirmed their participation in written form. The study methodologies were approved by the local ethics committee (#33/01) with a newly added addendum from 2024.

### 2.2 Analyses of CRC patients from public resources

The Limma R package (Ver 4.2.0) was used for differential expression analysis with an adjusted *P*‐value of 1e‐20 (for pairwise analysis) as a significance threshold. We applied the GEPIA2 database that covers the data from The Cancer Genome Atlas (TCGA) and Genotype‐Tissue Expression (GTEx) [13]. Box‐ ‐, violin‐ ‐, and scatter plots for selected differentially expressed genes (DEGs) were drawn using the TCGA and GTEx visualization website GEPIA2 [14].

### 2.3 Colon cell type

Analysis of bulk RNA‐seq datasets from 15 human organs, including colon, produced a cell type enrichment prediction atlas for all coding genes. The initial data was extracted from GTEx. The specific profiles across tissue types revealed 12 types of cells in colon by the Human Proteome Atlas (HPA; [15]), which cover 1918 genes. We performed the analysis for 7 main colon cell types: Colon enterocytes (369 genes), Colon enteroendocrine cells (338 genes), Enteric glia cells (240 genes), Mitotic cells in colon (85 genes), Endothelial cells (219 genes), Smooth muscle cells (166 genes), and Fibroblasts (42 genes). Additionally, 5 immunological cell types from colon are Macrophages (143), Neutrophils (65), Mast cells (29), T‐cells (108), and plasma cells (114).

### 2.4 Bioinformatics tools and statistics

#### 2.4.1 Statistical significance

Paired statistics for 2‐group analysis was based on 2‐tailed *t*‐test. Statistical significance was also computed using the non‐parametric Mann–Whitney U‐test. Kruskal–Wallis tests were used in single‐variable comparisons with more than 2 groups. Differences with *P* < 0.05 were regarded as statistically significant (unless mentioned otherwise). False discovery rate (FDR) was computed using the Benjamini‐Hochberg method. Hypergeometric distribution test was used to obtain *P*‐values for overlapping gene sets.

#### 2.4.2 Gene expression density plot

Conducted using RNA‐seq data from TCGA, combined with the Therapeutically Applicable Research to Generate Effective Treatments (TARGET) and the GTEx repositories using TNMplot [16].

#### 2.4.3 Enrichment tests

The database of gene sets from the Molecular Signatures Database (MsigDB). A collection of hallmark gene sets is a set of 50 main processes in cells with expert curation, with about 200 genes included in each hallmark set [17]. Testing overrepresentation analysis by slice representation. Different colored slices indicate the hallmarks (a total of 10) that are significant (using adjusted *P* < 0.05 as a threshold). The analysis used 6763 genes that are associated with any of the hallmarks as a reference set.

Enrichment for miRNAs was based on miRinGO, which addresses indirect gene target genes through transcription factors (TFs) according to miRNA expression in specific tissues [18]. Database of dbDEMC 3.0 (database of differentially expressed miRNAs in human cancers) covers 40 cancer types with large‐scale compilation of miRNA gene expression from experiments [19].

### 2.5 Interacting networks

Physical protein–protein interaction (PPI) set is based on IntAct with over 1 million  interaction datapoints. High confidence interactions were based on the molecular interaction (MI) score. The MI score is calculated on independent PPI evidence and complementary experimental methods. Spoke expansion refers to all pairs of bait‐identified interactions [20]. STRING network was used based on PPI confidence score (>0.6, or as mentioned). Connectivity network excluded neighborhood, gene fusion, and cooccurrence as evidence [21]. Network of miRNA‐genes TF regulation by miRNAs was extracted from EMT‐Regulome [22].

### 2.6 CRISPR‐Cas9 cell line screening

We used the precalculated correlation of dependency from DepMap using CRISPR/Cas9. CRISPR‐Cas9 screenings were reported for 19,144 genes across 1206 cell lines (primary and established), providing knockout fitness scores (14 days after transfection) [23]. Dependencies enriched in COAD were precalculated for ~1800 genes (identified by the DepMap CRISPR‐Cas9 project using the Public 23Q4 + Score, Chronos resource), with an expanded collection of cell lines and cancer types from BioGRID ORCS (Ver. 1.0.4) CRISPR screen [24]. We search for genes with correlated knockout fitness (called ‘co‐dependent’). A loss of fitness and a negative log fold change in the average representation of the relevant targeted sequence relative to the plasmid are indicative of the gene being essential.

### 2.7 Predictive analysis based on gene expression levels

KM Plot and ROC Plotter [25] were used to identify gene expression‐based predictive biomarkers for CRC that compiled publicly available datasets. By integrating gene expression data (RNA‐seq and Chip‐Seq) with chemotherapy, most genes were tested. A link of gene expression and therapy response using transcriptome‐level CRC data generates a ROC plot with detailed statistics on the relevance of any gene to therapy and clinical response [26]. To identify genes with altered expression in TCGA COAD samples in view of disruptive mutations in a gene set (5 genes), we applied the GENOTYPE module of MuTarget [27].

### 2.8 Establishment of COAD and healthy colon patient‐derived organoids

Detailed reagent information is provided in Text S1 Freshly resected tissue samples were transported on ice to the Institute of Pathology, where a pathologist confirmed tumor presence (for CRC samples) and processed healthy tissues. For healthy colon organoids, the mucosa was separated from the submucosa and muscularis, cut into 1–2 mm fragments, and washed three times in wash buffer (DPBS with 1% antibiotic–antimycotic solution and 0.1% gentamicin sulfate). Crypts were liberated via incubation in DPBS containing 2 mm Na‐EDTA for 1–2 h, followed by gentle pipetting and repeated collection of the crypt‐containing supernatant. Crypt solutions were then centrifuged (200 ***g***, 4 °C, 5 min) and washed twice.

To establish organoids from CRC tissues, small fragments were washed three times in the same wash buffer before enzymatic digestion with 5 mg·mL^−1^ Collagenase IV, 250 μL Dispase, and 1750 μL Basic Media (Advanced DMEM/F12, 1% Hepes, 1% Penicillin–Streptomycin, 1% Glutamax) in a shaking water bath at 37 °C for 15–30 min. Digestion was halted upon observing single cells and clusters, and the suspension was passed through a 100 μm cell strainer, centrifuged (300 ***g***, 4 °C, 5 min), and washed three times in wash buffer.

Isolated crypts or tumor cells were resuspended in Cultrex Basement Membrane Extract (BME) and seeded in 10 μL drops on prewarmed plates, which were incubated upside down at 37 °C for 30 min to allow polymerization. Organoids were maintained in Colon Expansion Media (Basic Media supplemented with 10% R‐spondin‐CM, 5% Noggin‐CM, 2% B27, 10 mm nicotinamide, 1.25 mm N‐acetylcysteine, 10 nm gastrin, 50 ng·mL^−1^ mEGF‐Rec, 500 nm A‐83‐01, 10 μm SB202190, 100 μg·mL^−1^ Primocin, and 1 μm PGE2). For healthy colon organoids, NGS‐Wnt recombinant protein (final 1 nm) was added [28]. Immediately after isolation and splitting, 10 μm Y‐27632 (Rho kinase inhibitor) was included in the expansion media. The study was approved by the Ethics Committee of the Medical Faculty, University of Magdeburg (#46/22).

### 2.9 PDO quality control by DNA analysis

Organoids were harvested, washed with DBPS, and cell pellets were frozen in liquid nitrogen and subsequently stored at −80°C until further use. Organoids were processed with the QIAamp DNA Mini Kit (Quiagen # 51306) following the manufacturer's protocol.

Targeted parallel sequencing was carried out using a Nextera Rapid Capture Custom Enrichment panel (Illumina, San Diego, CA, USA) covering all coding exons and flanking intronic sequence of ±20 nucleotides of target genes according to the manufacturer's instructions. Cluster generation and sequencing were implemented on an Illumina MiSeq System (Illumina). Reads were aligned and mapped to the human assembly hg19 (GRCh37). We used the varvis genomics software v.1.25.0 (Limbus Medical Technologies GmbH, Rostock, Germany) for SNV‐ and CNV‐analysis. A coverage depth of >100× has been reached.

This study exclusively used recently established primary patientsample–established cell models that were utilized for experiments right after NGS‐based malignancy verification, as mentioned above. Our recently published and rigorously enforced quality control measures for academic laboratories [29] ensure regular (after establishment at least once every three years) confirmation of unique genetic authenticity of in *in vitro* disease models using short tandem repeat (STR) assays, as previously described [30]. In addition, monthly conducted surveillance measures confirm the absence of bacterial contamination in cell cultures by employing commercial PCR‐based mycoplasma detection in culture supernatants.

### 2.10 Flow cytometric assessed surface expression and linked PDO metabolism

The importance of PDO for testing cellular physiology and drug screening was established (*e.g*., [31]). Single‐cell suspensions of PDOs (*n* = 5 from colon carcinoma, *n* = 2 from healthy donors) were prepared. Approximately 50,000 cells per sample were subjected to various staining protocols.

For the analysis of surface markers, cells were first incubated with a viability dye (Ghost Dye™ Violet 510, Cytek, Fremont, CA, USA) following the manufacturer's recommendations. They were then stained with the following fluorochrome‐conjugated antibodies for 30 min at 4 °C: PE‐Cy7 rat anti‐human CD44 (clone QA19A43, Biolegend, San Diego, CA, USA), FITC mouse anti‐human CD71 (clone M‐A712, BD Biosciences, San Jose, CA, USA), and CF647 rabbit anti‐human SLC7A11/xCT (polyclonal, biorbyt Ltd., Cambridge, UK).

Cystine uptake was assessed using BioTracker Cystine‐FITC Live Cell Dye (Merck, Darmstadt, Germany) at a final concentration of 0.5 μm. Cells were incubated for 30 min at 37 °C in 5% CO_2_ using serum‐free AIM‐V medium (Thermo Fisher Scientific, Waltham, MA, USA).

The labile iron pool (Fe^2+^) was measured using Phen Green SK diacetate (Cayman chemicals, Ann Arbor, MI, USA) at a final concentration of 10 nm. Cells were stained for 15 min at 37 °C in 5% CO_2_. Phen Green fluoresces in the absence of Fe^2+^, but its fluorescence is quenched by Fe^2+^, enabling semi‐quantitative analysis of iron levels.

All data was recorded on a NorthernLights spectral flow cytometer (Cytek, Fremont, CA, USA) after proper titration. Data were analyzed and visualized using FlowJo V10 (BD Bioscience). Exemplary gating strategy for cell surface epitope stainings, intracellular cystine and free ion pool can be found in Fig. S3.

### 2.11 Analysis of drug effect to PDOs by luminescence‐based quantification of cell mass viability

Organoids were expanded to a suitable size and underwent at least one passage post‐thaw before drug testing, typically between passages 8 and 15. For single‐cell seeding, organoids were harvested and washed twice in wash buffer (centrifugation at 250 ***g***) to remove debris, followed by a wash in DPBS (without Mg^2+^ and Ca^2+^). The pellet was then digested in TrypLE Express and incubated for 5 min in a prewarmed 37 °C water bath. After incubation, organoids were mechanically disrupted by pipetting, and dissociation into single cells was verified microscopically. If necessary, digestion was extended by an additional 5 min. The resulting single cells were filtered through a 40 μm cell strainer, counted, and resuspended in BME.

BME drops of 5 μL containing 2000 cells were seeded onto 96‐well plates and polymerized for 30 min at 37 °C in 0.5% CO_2_. Following polymerization, growth medium containing a Rho kinase inhibitor (Y‐27632) was added, and cells were cultured for 3 days to allow organoid formation. On Day 3, drug solutions were prepared at the desired concentrations and applied for 72 h. Viability was measured using a plate reader with luminescence detection (parameters: attenuation OD1, no lid, appropriate integration time). After 72 h of treatment, Cell Titer‐Glo 3D reagent was added, and viability was assessed following the manufacturer's instructions.

## 3 Results

### 3.1 Pairwise analysis of samples from colon cancer patients

**Fig. 1:**
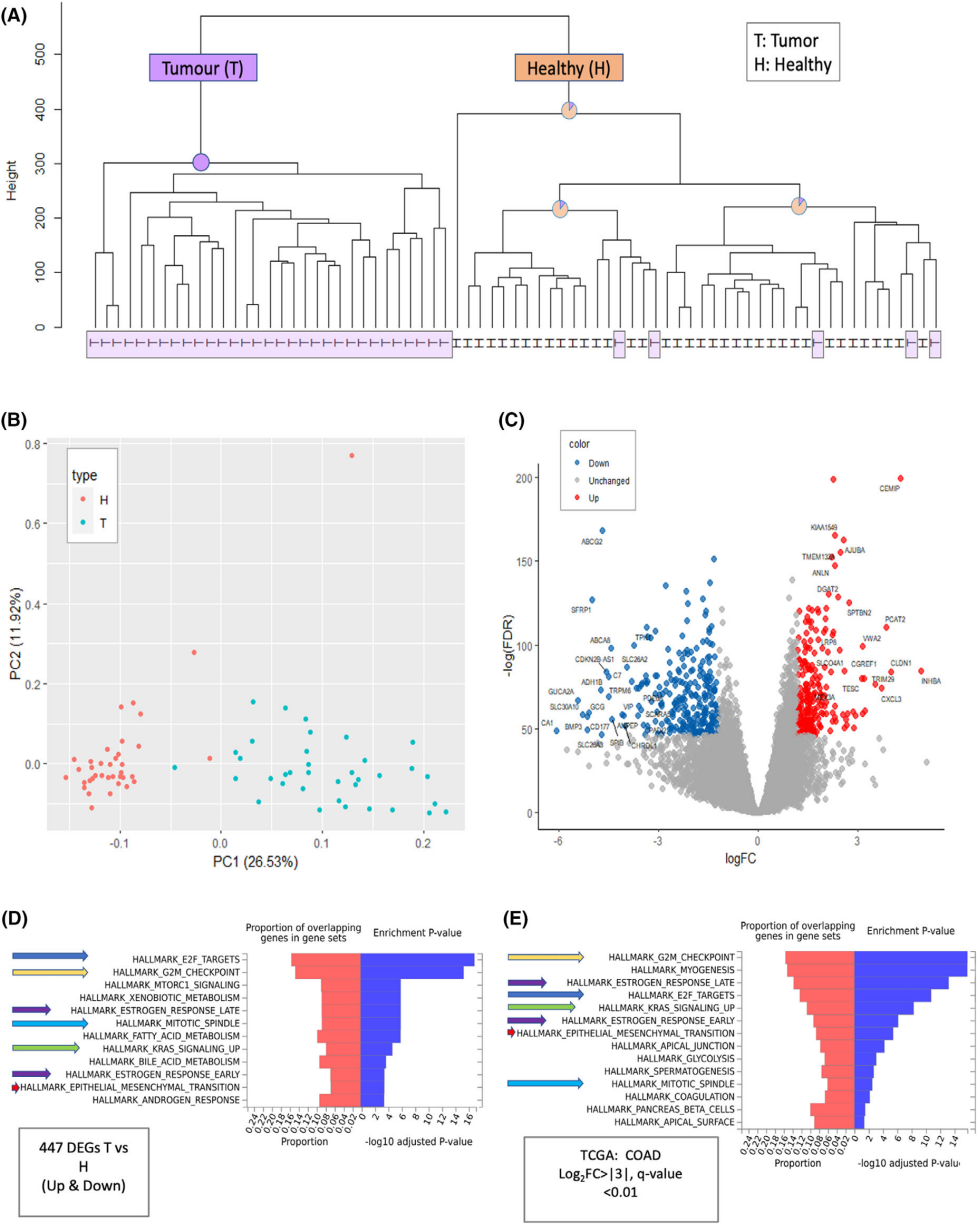
Analysis of the mRNA profiles from 32 colon cancer patients. (A) Unsupervised dendrogram of 73 samples from 32 participants. The main nodes are indicated by their purity for tumor (T) and healthy (H) colored purple and orange, respectively. The T samples in the dendrogram tree are highlighted with a light purple background. Samples for which more than two were collected from the same individual are marked with one or more asterisks, indicating the number of samples per individual. (B) PCA for 73 samples, based on the top 1000 differentially expressed genes (DEGs) colored by T and H in red and blue, respectively. The variance explained is indicated for PC1 and PC2. (C) Volcano plot representation of DEG analysis from RNA‐seq of T vs. H for the samples described in (A). Red and blue points mark the genes with significantly increased or decreased expression in T relative to H, respectively. Representative significant genes are indicated. (D) DEGs (up‐ and downregulated; 477 genes) were tested for enrichment against Hallmark gene sets from MSigDB. (E) Overlap of enriched Hallmark pathways with external GEPIA2 data (COAD; |log_2_FC| > 3, *q* < 0.01) as indicated with same‐color arrows in each figure panel, confirms shared cancer‐related signatures.

Figure 1A shows the unsupervised partitions of all 73 samples labeled tumor and healthy (T and H, respectively). The dendrogram shows a clear partition of all samples into two main branches. The tumor (T) branch is 100% consistent (purple, 31 samples), and the second major branch is mostly composed of healthy samples (88% of 42 samples, orange), with only 5T‐labeled samples clustered with the H samples. Fig. 1B shows that dimensional reduction by principal component analysis (PCA) supports a successful unsupervised partition of T and H samples, with 37% of the total variance explained by PC1 and PC2. The PCA used the top 1000 ranked differentially expressed genes (DEGs). A similar successful partition of T and H was achieved by PCA, whose input consisted of the entire RNA‐seq profiles (a total of 13,682 identified transcripts).

Next, we analyzed the consensus DEGs from all patients. Each patient was analyzed with respect to their own health‐labeled sample. Single samples from the tumor and healthy tissue were normalized and compared internally (according to the number of samples available). Altogether, we performed global analyses of 32 pre‐analysis patients to confirm high statistical significance and a minimal fold change threshold per gene. Specifically, the analysis was restricted to genes with a minimal statistic of FDR *P*‐value <1e‐20, with a minimal average expression of 10 counts per million (CPM) and limited the analysis to coding genes (*i.e*., 92% of all mapped transcripts). Such filtration reduced the 13,682 unique gene transcripts to 9045 genes that were further analyzed (Fig. 1C).

To investigate the functional significance of our RNA‐seq data, we analyzed the expression profiles in the context of the Hallmark gene sets from MSigDB. For clinical relevance, it is essential to focus on consistent expression differences in T to H samples. We reanalyzed DEG at a relaxed threshold. For enrichment of cancer hallmarks, DEGs were selected with FDR ≤1e‐20, a minimal fold change of 2.3 (*i.e*., log(FC) ≥ |1.2|), and a minimal average expression of 10 CPM (Fig. 1D, Table S3).

To validate our findings, we compared the enrichment results with external data from GEPIA2, using the COAD dataset and applying stringent criteria (|log_2_FC| > 3, *q*‐value < 0.01) (Fig. 1E). Our analysis revealed a substantial overlap in hallmark gene sets enriched in both datasets, supporting the robustness of our findings. Key cancer‐associated pathways, including G2/M checkpoint regulation, E2F targets, mitotic spindle assembly, and estrogen signaling, were consistently enriched and highly significant (as indicated with same‐colored arrows in each subpanel).

### 3.2 Colon cancer DEGs reveal hallmarks of cell cycle and metabolic program in cancer samples

Major cellular biological processes (see Section 2) cover the preselected 50 gene sets (MSigDB). Table 1 lists the most enriched hallmark sets (Adjusted *P*‐value <1e‐05). Several observations can be made based on the results in Table 1. Firstly, the strongest enrichment is for a set of genes encoding cell‐cycle‐related targets of E2F transcription factor and G2/M checkpoint, which are exclusively composed of upregulated genes. Moreover, for the most significantly enriched hallmarks, the associated genes were consistent in their expression trend (i.e., either up‐ or downregulated). An exception is a hallmark called ‘estrogen response‐late’ that shows a mixture of up‐ and downregulated genes. Lastly, the metabolic hallmarks are primarily associated with downregulated genes. For a list of enriched hallmarks along with their associated genes (total 27 sets), see Table S4.

**Table 1:** Enrichment analysis of DEGs for the set of MSigDB hallmarks.

Hallmarks (H) gene set (MsigDB)	N in geneset	# up	# down	Adj. *P*‐val
H: E2F_TARGETS	194	30	0	6.93E‐18
H: G2M_CHECKPOINT	192	28	0	2.87E‐16
H: MTORC1_SIGNALING	193	15	2	8.65E‐07
H: XENOBIOTIC_METABOLISM	196	4	13	8.65E‐07
H: ESTROGEN_RESPONSE_LATE	196	9	8	8.65E‐07
H: MITOTIC_SPINDLE	197	15	2	8.65E‐07
H: FATTY_ACID_METABOLISM	155	2	13	9.38E‐07
H: KRAS_SIGNALING_UP	195	11	4	1.56E‐05

**Fig. 2:**
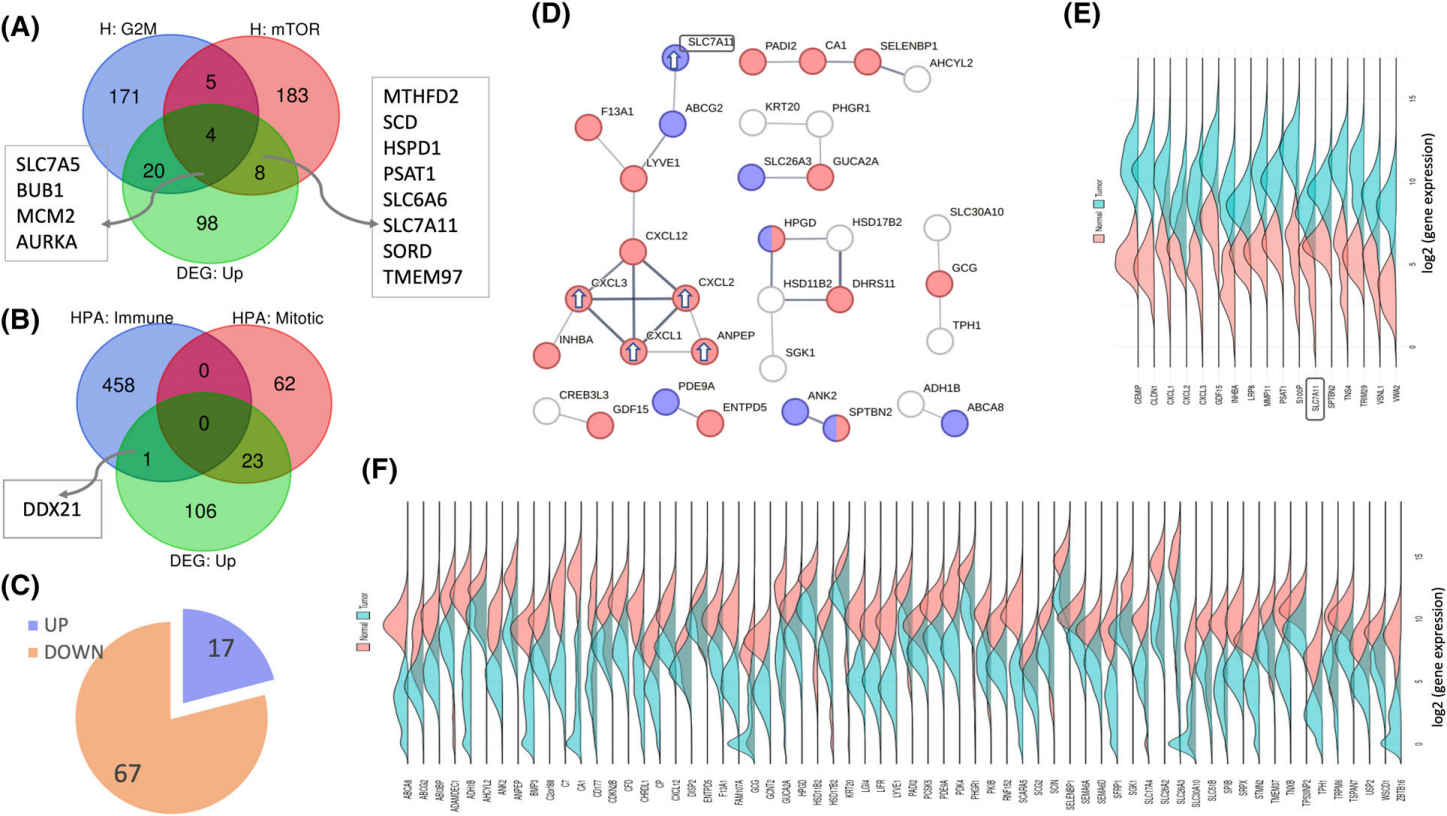
Upregulated DEGs and cellular processes. (A) Venn diagram of the upregulated DEGs (130 genes) and the cancer hallmark sets of ‘cell cycle G2M checkpoint’ and ‘mTORC1 signaling’. The overlapping genes are listed. (B) Venn diagram of the upregulated DEGs (130 genes) and the colon cell types (see Section 2). (C) Pie chart of the top 84 DEGs filtered by a threshold of FC ≥|5|, and partitioned into up‐ and downregulated genes. (D) STRING‐based network (confidence threshold 0.5). Colored are genes that are annotated by GO cellular component annotation (GO_CC) as extracellular and plasma membrane regions (red and blue, respectively). The upregulated genes in the largest connected component are indicated by white arrows. The SLC7A11 gene is highlighted. (E) Density plot analysis of the 17 upregulated DEGs (alphabetic order). Expression density plots of healthy and tumor samples are in pink and blue, respectively. The SLC7A11 gene is marked. (F) Density plot analysis of the 67 downregulated DEGs (in alphabetic order).

The results from Table 1 can be broadly classified into two larger themes: cell‐cycle‐related (*e.g*., mitotic spindle, G2M checkpoint, and genes encoding cell cycle E2F) and nutrients and metabolic programs (*e.g*., fatty acids degrading, genes involved in processing drugs and other xenobiotics). Fig. 2A tests gene overlap of the hallmark sets that belong to these main themes, with 20 upregulated genes overlapping the hallmark set of cell cycle, 8 upregulated genes signifying mTOR signaling (including SLC7A11), and 4 upregulated genes overlapping both sets.

### 3.3 Upregulated genes identify a subpopulation of mitotic cells

The colon is a complex tissue composed of numerous cell types. We tested the set of most significantly upregulated genes (total 130) with respect to the 12 characterized cell types that are signified by enriched gene sets, based on the Human Proteome Atlas (HPA) classification (Table S5). Only a significant overlap was detected with the mitotic cell type, with 23 DEGs overlapping 85 mitotic cell enriched genes (enrichment *P*‐value 3.5e‐05) (Fig. 2B). Among these genes are kinesin‐like proteins that act in chromatid segregation (KIF2C, KIF14, KIF20A), genes that participate in the cell cycle via DNA repair mechanisms (RAD51AP1, EXO1, BRCA2), and checkpoint control genes that act in DNA replication (TPX2, BUB1, NUF2, CDC6).

Notably, among the colon‐centric immune‐associated cell types (total 459 genes, see Section 2), only DDX21 (DEG *P*‐value FDR 5.30e‐46) was detected (Fig. 2B). In colon cancer, the knockdown of DDX21 inhibited cell growth by activating CDK1. DDX21 was postulated to mediate this effect via chromatin modulation of the CDK1 promoter [32]. CDK1 was also identified among the upregulated genes overlapping with the mitotic signature. Colon cell types (1918 genes, 12 cell types; see Section 2) are listed in Table S5.

### 3.4 Enrichment of extracellular and plasma membrane protein‐encoding genes among the strongest DEGs

We then tested the possibility of identifying tumor vs. healthy genes and focused on the subset of genes showing the most extreme differential expression signals. To this end, we analyzed a subset of 84 DEGs with fold change (FC) of >|5|. While this is an arbitrary threshold, it captures the maximally responsive DEG group. Fig. 2C shows that at this threshold, 80% of the genes were downregulated and only 20% were upregulated.

Figure 2D shows a connectivity map of these DEGs (STRING confidence score ≥ 0.5; minimum ≥ 2 connected genes). The largest subnetwork (10 nodes) included the SLC7A11 transporter, genes that function in mitochondria, and a set of secreted cytokines that included upregulated genes (marked with arrows). The other subgraphs include the downregulated genes. Most connected genes (70%) were assigned to either extracellular regions (GO cellular component, *P*‐value = 3e‐06; colored red) or the plasma membrane region (*P*‐value = 0.003; colored blue). Notably, there were 6 transporters among the 84 upregulated genes, with SLC7A11 being the most strongly upregulated (Table S3). These findings highlight the importance of plasma membrane signaling and extracellular communication.

To validate that the DEGs from our study are in agreement with the large‐scale available data from cancer resources, we performed density plot analysis for the upregulated (Fig. 2E) and downregulated (Fig. 2F) genes with data extracted from TCGA. The results show a complete agreement in the list of all 84 DEGs (Table S3) regarding the expression level trends in healthy and tumor samples of our CRC cohort.

### 3.5 SLC7A11 and its interactors exhibit a coordinated upregulated expression in CRC

**Fig. 3:**
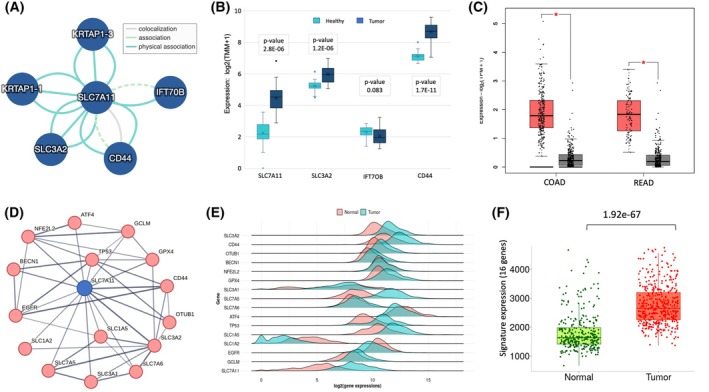
Signature of SLC7A11 network in colon cancer. (A) Direct physical associations of SLC7A11 (IntAct with MI score >0.5). Each partner was identified by interaction through multiple Section 2. Dashed edge marks spoke expansion (see Section 2). (B) Box plot of the 32 CRC patients with the expression of a subset of genes from (A). (C) Box plot of log expression measured by transcripts per million (TPM) for SLC7A11 from TCGA for COAD (461) and rectum adenocarcinoma (READ, 172) samples. Tumor and healthy are colored red and gray, respectively. (D) Interacting genes centered by SLC7A11 according to STRING (confidence score >0.6), limited to the most reliable PPI connectivity (total 16 genes). (E) Density plots for 16‐core genes from (A). Healthy and tumor samples are marked in pink and blue, respectively. (F) Box plot for the signature of all SLC7A11 16‐core gene set for healthy (normal, green) and tumor (red). Each dot represents a datapoint from COAD samples. Error bars = SD; statistical significance in (B, C, F) was determined using an unpaired two‐tailed Student's *t*‐test.

The enrichment of metabolic hallmarks (Table 1), and the results of Fig. 2 indicating the plasma membrane enrichment, led us to focus on the Xc‐system, consisting of SLC7A11 (xCT) and SLC3A2 (heavy chain 4F2hc). This transport system governs the intracellular redox balance in cancer cells. Fig. 3A lists validated direct partners of SLC7A11 (see Section 2), along with the expression levels of these directly interacting partners in healthy (H) and tumor (T) samples for all 32 CRC patients (Fig. 3B). The KRTAP1‐1 and KRTAP1‐3 were below the expression threshold in CRC samples. The IFT70B (also called TTC30B) was poorly expressed in CRC samples and showed no difference between expression in T and H. These genes were not further analyzed. SLC7A11, SLC3A2, which compose the Xc system, and CD44, that stabilizes SLC7A11 on the cell surface, were significantly increased in T vs. H. To generalize and validate our findings, we compared the expression trend of SLC7A11 among publicly available COAD (461) and READ (172) samples from TCGA and confirmed the significant overexpression of SLC7A11 transcripts in T vs. H (Fig. 3C).

The set of genes that were strongly connected to SLC7A11 across multiple tissues was further analyzed (STRING confidence score ≥0.7; coined SLC7A11‐core set, 16 genes). SLC7A11 PPI displays PPI association with SLC3A2, CD44 (direct, as in Fig. 3A) but also with additional transporters (6) from the SLC family. Notably, the expression profiles of SLC genes with regard to the metabolic demands in cancer tissues suggest their link to the tumor immune microenvironment (TME) and drug response [33]. Among the SLC7A11‐core set, there are other metabolic genes. For example, OTUB1, a specific deubiquitylating enzyme with a cysteine protease activity, and BECN1 (Beclin 1) that regulates vesicle trafficking, autophagy, and apoptosis. Other core genes (ATF4, NFE2L2, and GPX4) are activated by starvation, oxidation, and ER stress. Specifically, the transcription factor (TF) ATF4 induces various amino acid transporters and enzymes that determine the metabolic state of cells (*e.g*., redox balance, energy production, nucleotide synthesis). Other core genes are directly associated with cancer progression (TP53 and EGFR) and drive cell migration, differentiation, and cell growth (Fig. 3D).

Figure 3E shows a density plot for all 16 core‐SLC7A11 genes according to COAD expression levels from TCGA. The expression levels of most core genes are higher in tumors relative to healthy samples. However, SLC1A2, EGFR, and ATF4 display an opposite expression trend. The analysis for all 16 SLC7A11 core genes among our 32 CRC cohort is shown in Fig. S1. We have confirmed that the overall signature of all 16 core‐SLC7A11 genes remains highly significant for distinguishing tumor from healthy tissue for the entire TCGA cohort of COAD (Mann–Whitney *P*‐value 1.9e‐67, Fig. 3F). We concluded that the SLC7A11‐core set is a hub for processes of mitotic cells and metabolic balance in our CRC patients, and this signal is validated across 630 samples from TCGA (COAD and READ).

### 3.6 Knowledge‐based inspection of SLC7A11 determines its oncogenic potency

**Fig. 4:**
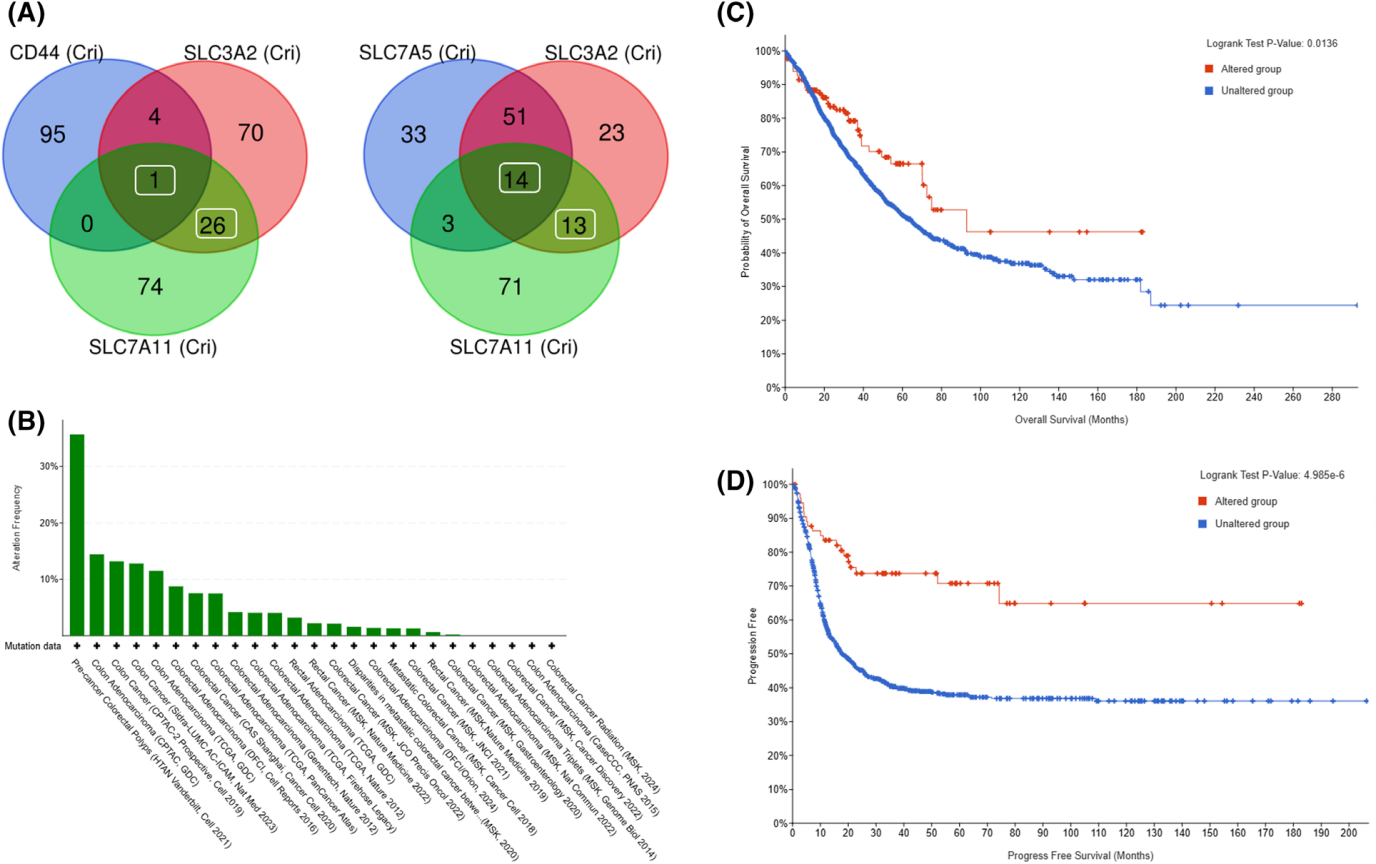
Gene set of CRISPR‐induced Xc‐ fitness and CRC survival. (A) Venn diagram for codependent genes by CRISPR‐Cas9 setting (DepMap‐based, see Section 2). The top 100 correlated genes were included in the analysis. There are 27 shared genes (left, white frame) between the two membrane transporters, with only 1 overlapped gene also with CD44. In contrast, the SLC7A5 displayed a strong shared signal with SLC3A2 (Right, 65 genes); among them, 14 genes are shared by all three gene sets. (B) A total of 17 bowel cancer studies from cBioPortal (a total of 8041 samples) were selected. From these studies, we focused only on samples with mutations (while excluding samples with structural variations) in their genes. Specifically, we included only samples with truncating mutations, which further included nonsense, frameshift (frameshift insertion, frameshift deletion), nonstart, nonstop, and splice mutations. Selected studies had ≥100 samples each (the appendiceal cancer cohort was excluded). The survival plots (C and D) indicate the unaltered and altered sets (blue and red, respectively) for samples with alteration in any of the 27 shared genes (shown in A). (C) The overall survival plot (OS) for 300 months. (D) Progress‐free survival (PFS) curve for 220 months. Statistical significance is indicated. Statistical significance in (C, D) was determined using the log‐rank test.

We sought to identify a functional network of SLCA11‐correlated genes by considering gene perturbations in a cellular context. To this end, we tested the essentiality, specificity, and efficacy of CRISPR dependency screens. The top co‐dependent genes by CRISPR‐Cas9 setting specify the degree of gene essentiality and replication fitness (see Section 2). To further inspect the importance of SLC7A11 in colon cancer, we investigated the CRISPR‐based dependency map for the Xc‐ system (*i.e*., SLC7A11, SLC3A2) and the CD44, which stabilizes the Xc system in the plasma membrane. We compared genes that were characterized by their correlation following CRISPR gene depletion and focused on the top 100 listed correlated genes for each. Fig. 4A compared genes that by their overlap among the co‐dependent CRISPR screening results. There are 27 genes that are shared by the Xc system. As expected, the cell surface glycoprotein CD44 involved in cells' interactions, adhesion, and migration, shows a minimal overlap due to its unique functionality. Only SDHA is shared among all three components (including CD44). This gene is a subunit of complex II in the mitochondrial respiratory chain, and it highlights the importance of mitochondrial function as a central hub for Xc‐ gene dependency. In contrast, a substantial overlap between the co‐dependent SLC7A5 and SLC3A2 sets was observed (63 genes). The detailed results of the Venn analysis are available in Table S6.

To test the impact of alterations in the overlapping genes (27) on the survival of COAD patients, we created a cohort composed of 17 bowel‐derived complementary cancer studies. From these studies, we focused only on samples with mutations of specific types: truncating mutations, which include nonsense, frameshift (frameshift insertion, frameshift deletion), nonstart, nonstop, and splice mutations. About 4.2% of all samples (313 samples) carry these specific mutations in any of the 27 shared gene sets (Fig. 4B, Table S4). Overall survival (OS, Fig. 4C) and progression‐free survival (PFS, Fig. 4D) show that for both survival settings, the survival of the altered genes is markedly enhanced. A hazard ratio (HR) indicates an improved survival relative to the unaltered group. The results are consistent with the presence of an overexpressed level of SLC7A11 in tumors, where a reduction in SLC7A11 expression and its correlated gene set are linked to improved survival. This is the basis for the importance of SLC7A11 inhibitors in therapy [16].

### 3.7 Functional analysis of gene dependencies in the Xc system using *in silico* approach

**Fig. 5:**
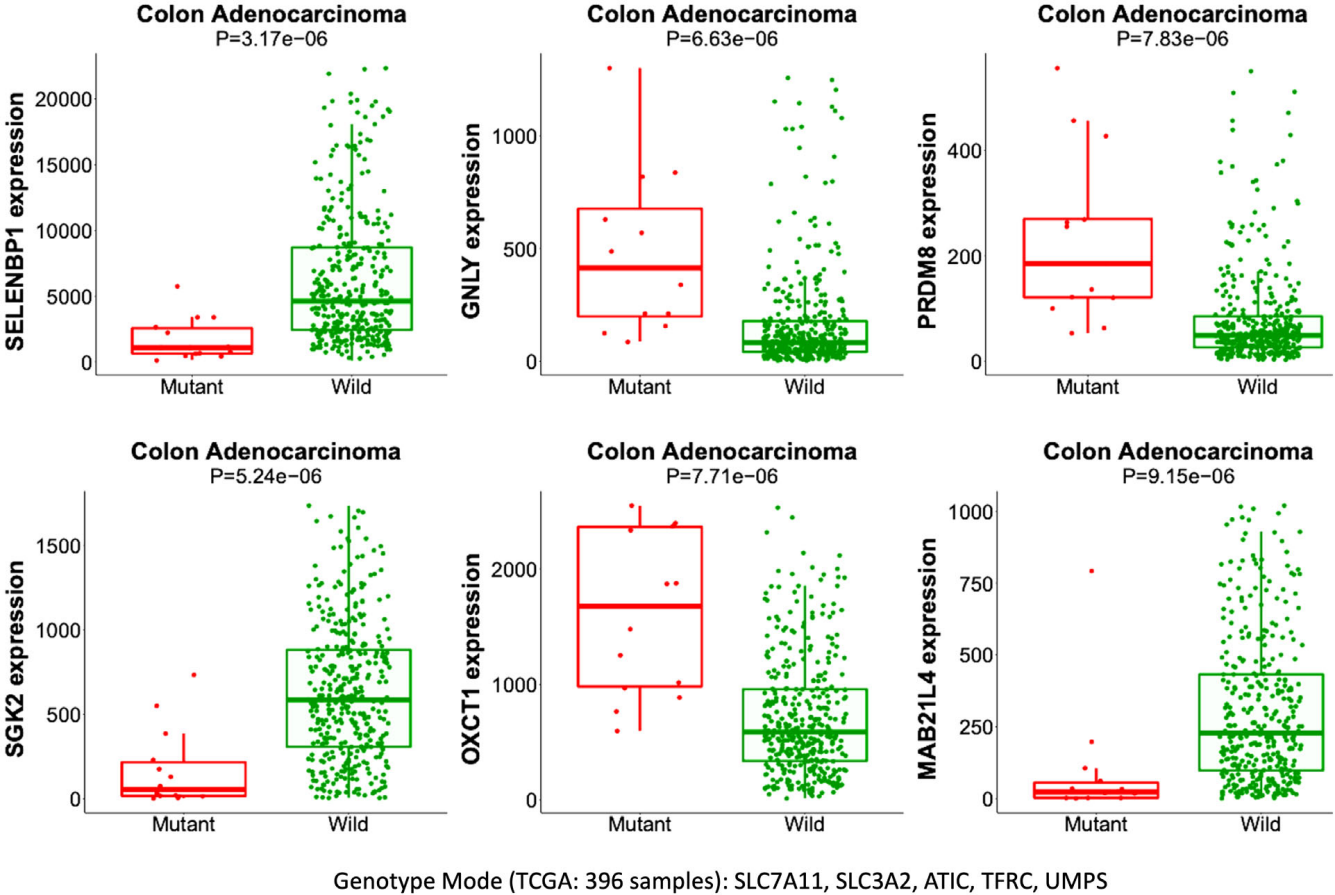
Analysis of MuTarget for paired genomic and transcriptomic data from COAD TCGA samples. Genotype mode. Six most significant altered gene expression related to the combined damaging mutations in SLC7A11, SLC3A2, ATIC, TFRC and UMPS (14 samples). Only significant changes of individual gene expression were considered (FC≥|2|, *P*‐value <1e‐3, minimal ≥100 CPM). Error bars = SD, The reported *P*‐value is based on Mann–Whitney U‐test.

To further substantiate a functional link between the strongly increased gene expression in COAD and robust results from CRISPR codependency tests (Table 2), we looked for genes whose expression is altered with respect to the loss of function (LOF) of selected gene sets (using MuTarget, see Section 2, Fig. 5A). The logic of this analysis is to provide a more direct connection between the damaged genotype and the expression of overlooked genes as an outcome. Note that the MuTarget approach is indifferent to the expression trend in tumors vs. healthy samples. We restricted the analysis to the 5‐gene functional set of SLC7A11, SLC3A2, ATIC, TFRC, and UMPS (coined FunSet, Table 2), only with disruptive damaging mutations. We sought to find genes that were significantly changed in their expression (up‐ or down‐modulated) for the genotype‐based partition of COAD samples. The results of the six most significant genes are shown in Fig. 5. In the collection of samples where SLC7A11 and its associates lost their function due to mutations, SELENBP1 and SGK2 were downregulated, while OXCT1 was upregulated. While causality cannot be inferred from such analyses, the affected genes are all implicated in metabolic regulation and epithelial–mesenchymal transition (EMT). Specifically, OXCT1 is a mitochondrial matrix enzyme that plays a central role in ketone catabolism. SELENBP1 is a selenium‐binding protein that may be involved in sensing reactive xenobiotics, and SGK2 is a serine/threonine‐protein kinase that is involved in the regulation of membrane ion channels and amino acid transporters, both associated with their impact on EMT [34, 35] (Fig. 5). The results for MuTarget identified genes are in Table S7. Whether such gene targets specify the contribution of EMT in CRC calls for further investigation. We performed a GO annotation enrichment analysis, linking changes in gene expression to the corresponding FunSet. The enriched annotations among the upregulated genes supports the occurrence of immune response genes—for example, GO_BP, adaptive immune response (*P*‐value 4.4e‐34) and antigen processing and presentation of peptide (*P*‐value 9.2e‐53). In contrast, the annotations of the downregulated genes (*e.g*., SGK2) are linked to various cell homeostasis processes, negative regulation of Wnt signaling, ion transport, xenobiotic metabolic process, and more (see Table S7). We conclude that via such COAD sample stratification, candidate genes and pathways could lead to drug targeting and molecular manipulation of overlooked targets.

**Table 2:** Overlapping CRISPR codependent genes of Xc‐system genes in COAD samples. FC is the fold change.

Gene	Description	Main function	FC T vs. H^a^	FC met. vs. T	FC met. vs. H^b^
KRT16	Keratin, type I cytoskeletal 16	Intermediate filament	**13.13**	0.13	1.73
SLC7A11	Cystine/glutamate transporter	Cystine transport as redox regulator	**9.22**	0.34	3.15
PRH2	Proline‐rich protein HaeIII subfamily 2	Secreted glycoprotein	**3.27**	0	0
MTHFD1	Methylenetetrahydrofolate dehydrogenase	*De novo* purine synthesis	**2.72**	0.62	1.7
ATIC	Formyltransferase/IMP cyclohydrolase	*De novo* purine biosynthetic pathway	**2.7**	2.15	**5.79**
RPIA	Ribose 5‐phosphate isomerase A	Pentose‐phosphate pathway	**2.65**	0.77	2.03
UMPS	Uridine monophosphate synthetase	*De novo* pyrimidine biosynthetic pathway	**2.65**	6.34	**16.79**
CAD	Transcarbamylase‐dihydroorotase	*De novo* biosynthesis of pyrimidine nucleotides	**2.62**	1	2.61
TFRC	Transferrin receptor	Cellular iron uptake	**2.51**	1.91	**4.79**
RAB36	Ras‐related protein Rab‐36	Vesicle‐mediated transport	**2.47**	0.8	1.98
SLC3A2	4F2 Cell surface antigen heavy chain	Transport of L‐type amino acids	**2.27**	0.54	1.22

a In boldface FC >2.0 for Tumor (T) vs. healthy (H).

b In boldface genes that amplified the metastatic (Met.) state.

### 3.8 Differential protein expression of xCT and its interactors determines chemosensitivity and ferroptosis in PDO models

**Fig. 6:**
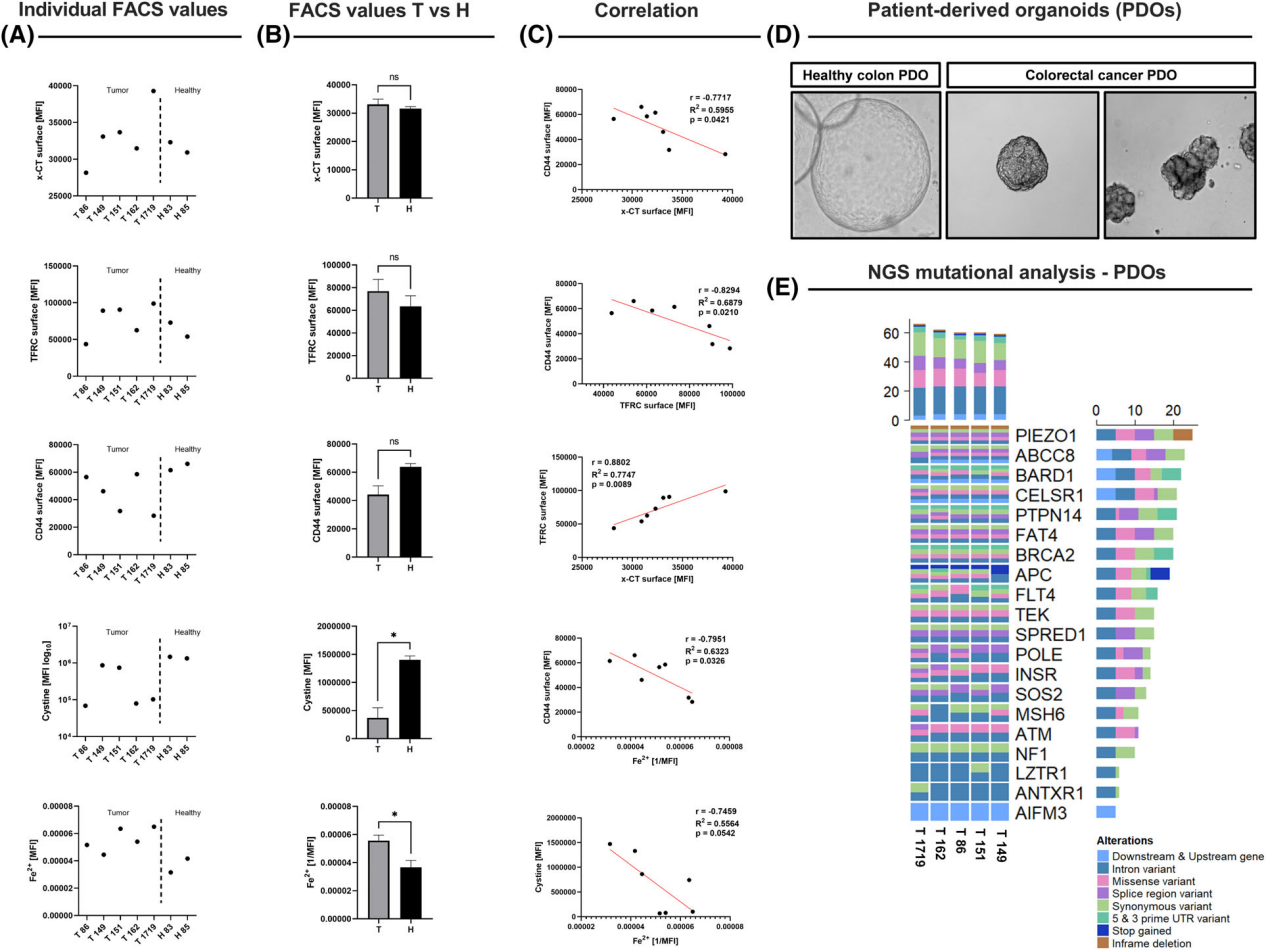
Functional assays using flow cytometric analyses of the protein levels of xCT, TFRC, CD44, and the relative amounts of cystine and Fe^2+^ in tumor vs. healthy PDO samples (A, B) and their correlations (C) using simple linear regression analysis. Unpaired *t*‐test was used to determine statistical significance between T & H groups in (B) with error bars indicating SEM. T = tumor PDO (*n* = 5), H = healthy PDO (*n* = 2). (D) Exemplary images of used PDOs showing different distinct morphologies, with NGS profiles illustrating key mutational landscapes in representative PDOs (E). *P‐val <0.05; **P‐val <0.01; ***P‐val <0.001; ****P‐val <0.0001.

To validate CRISPR‐based functional data, we analyzed newly generated CRC patient‐derived organoids (PDOs) to assess the expression of xCT‐interacting proteins. This included metabolic profiling and drug response assays targeting xCT inhibition under standard treatment regimens. These tests were performed in PDOs confirmed for genetic fidelity and morphologic accuracy (Fig. 6D,E). Clinical parameters of the PDOs used in this study are listed in Table 3.

**Table 3:** Clinical parameters of patients with CRC and basic molecular properties of retrieved tissue specimen propagated for *in vitro* model generation.

Tumor code	Primary/metastasis	Gender	Age at surgery	TNM	Grading	MMR	Mut
86	Primary	Male	59	pT4a pN0 M0 L0 V0 Pn0 R1	G3	MSS	N.A.
149	Metastasis	Female	78	pT4b pN2a pM1 L1 V1 Pn1 R1	G3	N.A.	N.A.
151	Primary	Male	74	pT3 pN1b M0 L1 V0 Pn0 R0	N.A.	MSS	N.A.
162	Metastasis	Male	54	pT3 pN2b pM1 L1 V1 Pn1 R1	G3	MSS	KRAS
1719	Primary	Female	60	pT1 pN0 M0 L0 V0 Pn0 R0	Gx	MSS	N.A.

Flow cytometric analyses revealed no statistically significant differences in xCT surface expression between tumor and healthy colon organoids, despite slightly elevated mean fluorescence intensity (MFI) values in tumor organoids (Fig. 6A,B). CRC‐derived PDOs demonstrated significantly lower intracellular cystine concentrations and higher levels of unbound Fe^2+^ compared to healthy organoids (*P*‐val = 0.018, *P*‐val = 0.039, respectively). Although not directly tested in this study, the elevated cystine levels in healthy PDOs may enhance their protection against ROS by subsequently enriching higher GSH levels, while their reduced Fe^2+^ content suggests diminished susceptibility to ferroptosis. Interestingly, no correlation was observed between xCT expression and intracellular cystine levels (*r* = −0.13, *P*‐val = 0.78). However, xCT expression showed a strong and very significant positive correlation with the expression of transferrin receptor (TFRC) levels (*r* = 0.88, *P*‐val = 0.009) and, unexpectedly, a significant negative correlation with the expression of CD44 (*r* ≈ −0.77, *P*‐val = 0.042). Additionally, CD44 inversely correlated with intracellular Fe^2+^ levels (*r* = −0.8, *P*‐val = 0.0326), suggesting a potential antagonistic relationship between this marker and iron accumulation (Fig. 6C). We concluded that functional analysis using PDOs allows for a closer view of the tumor pathophysiology and person‐to‐person heterogeneity.

**Fig. 7:**
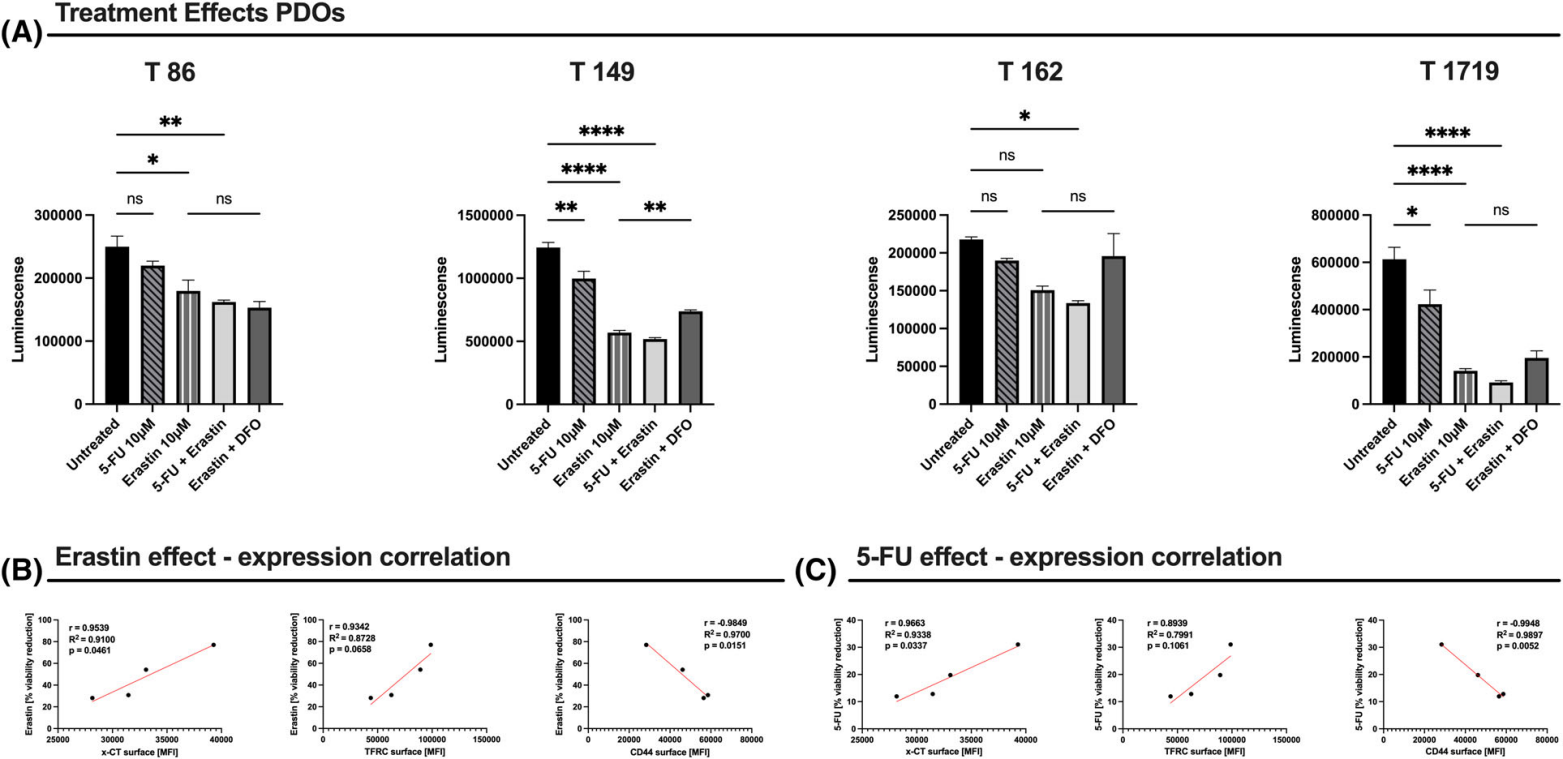
Luminescence‐based viability of patient‐derived tumor organoids (*n* = 4) after treatment with 5‐FU 10 μm and the ferroptosis inducer Erastin 10 μm, either alone or in combination, with or without the iron chelator DFO 100 μm (A). Ordinary one‐way ANOVA was used to determine statistical significance between treatment regimes, with error bars indicating SEM. Panels (B) and (C) show correlations by simple linear regression analysis between the baseline expression (MFI) of xCT, TFRC, or CD44 and the percentage viability reduction under Erastin‐ or 5‐FU‐based treatments. The *P*‐value thresholds are: *P‐val <0.05; **P‐val <0.01; ***P‐val <0.001; ****P‐val <0.0001.

Drug viability assays on tumor organoid lines (T86, T162, T149, T1719) revealed differential responses to treatment with the standard of care compound 5‐fluorouracil (5‐FU; 10 μm), Erastin (10 μm), or their combination (Fig. 7A). While T86 and T162 exhibited minimal viability reduction (*P*‐val < 0.05, *P*‐val > 0.05), T149 and T1719 were highly susceptible to all treatments, with partial rescue observed upon co‐administration of Erastin (blocks the activity of system Xc^−^) and the iron chelator deferoxamine (DFO) (T149, T162, T1719). Correlative analyses demonstrated that PDOs with elevated xCT surface expression were more sensitive to Erastin (*r* ≈ 0.95, *P*‐val = 0.046) (Fig. 7B) and exhibited enhanced sensitivity to 5‐FU (*r* = 0.97, *P*‐val = 0.034) (Fig. 7C). Elevated TFRC levels were associated with increased sensitivity to Erastin (*r* ≈ 0.93, *P*‐val ≈ 0.0658), whereas higher CD44 expression correlated negatively with both Erastin efficacy (*r* ≈ −0.98, *P*‐val = 0.015) and 5‐FU‐induced cytotoxicity (*r* ≈ −0.99, *P*‐val = 0.005).

Collectively, these findings highlight the differential roles of xCT, TFRC, and CD44 in modulating drug responses in CRC PDOs. Collectively, our results show that differentially expressed xCT, TFRC, and CD44 are able to predict chemotherapy treatment response in a correlative manner. The marked vulnerability of T149 and T1719 organoids to Erastin‐ and 5‐FU‐induced cell death underscores the therapeutic potential of targeting these pathways.

## 4 Discussion

Aberrant activation of SLC7A11 is involved in various cancer types, where it influences redox balance, metabolic flexibility, immune function, and ferroptosis [36]. We have focused on the role of the Xc‐ system in CRC cancer. Our findings revealed a distinct and CRC‐specific DEG cluster, including SLC7A11, that links overlapping genes from mitotic cell signatures to x‐CT‐specific cell metabolism (Fig. 2B). SLC7A11 may have different effects on different cancer cell types. For example, in glioblastoma, the overexpression of the Xc‐ system induces oxidative stress and apoptosis, in contrast to CRC, where it is a strong mediator of cell viability. Clinically, suppression of SLC7A11 function in CRC (*e.g*., by p53 or BECN1) can activate ferroptosis, which makes the tumor sensitive to radiotherapy. To further analyze the cellular role of Xc‐, we inspected the downstream glutathione pathways (*e.g*., GPX4, GPX8). We observed that the expression levels of GPX genes remained stable within the cohort of 32 patients (Table S2). Essential and co‐essential genes across many cell lines are a useful approach to identifying shared functional pathways [37]. The CRISPR‐based platform for GPX4 and GPX8 across 39 cell lines that originated from COAD did not support such a coherent signature of codependency. These results argue for the involvement of alternative pathways for Xc‐ system driven tumorigenesis. In contrast, SLC7A5 was exceptionally correlated with SLC3A2 (Fig. 4A). The role of SLC7A5 in CRC is to maintain intracellular amino acid levels following KRAS activation through transcriptional and metabolic reprogramming [38].

Among the genes that strongly correlate with the Xc‐ system in CRISPR‐based assays, TFRC (transferrin receptor) was found to be upregulated in COAD and implicated in promoting metastatic tumors (Table 2). Due to its role in iron accumulation and activation, TFRC also promotes nucleotide biosynthesis, DNA repair, and cell survival in colorectal tumors [39]. Additionally, impairing TFRC function has been suggested as a target for inhibiting tumor growth. Specifically, reducing the iron influx can induce DNA damage and apoptosis [40]. The interplay between TFRC and SLC7A11 provides cancer cells with a survival advantage based on these findings. We suggest that altering iron homeostasis and redox balance through manipulating the levels of TFRC and SLC7A11 can serve as potential therapeutic targets.

To further elucidate the functional role of SLC7A11 in COAD, we employed a short‐term cultured PDO platform. Quality control of the generated PDOs revealed typical COAD‐specific mutation profiles (Fig. 6D,E) and allows comparison between *in vitro* and clinical drug responses as a function of the relevant driver mutations. A recent study by De *et al*. [41] highlights the potential of targeting patient‐specific KRAS G12D mutations to promote ferroptosis, particularly when combined with other ferroptosis inducers. This approach strengthens the clinical relevance of xCT as a therapeutic target and underscores the importance of tailoring treatment strategies to individual mutation profiles. Our results add new knowledge proposing a particular centrality of xCT activity in CRC by demonstrating how differential xCT cell surface expression can predict therapy responses to different treatment regimes. Consistent with previous studies showing that xCT regulates redox balance and ferroptosis in various malignancies, our data reveal a paradoxical finding. CRC organoids displaying higher xCT expression exhibited higher sensitivity to 5‐FU, contradicting the conventional view that xCT upregulation confers chemotherapy resistance. A plausible explanation for this discrepancy may lie in the heightened metabolic demands and redox flux imposed by xCT overexpression. Under certain conditions, this pro‐survival mechanism can render cells more vulnerable to genotoxic or ferroptosis‐inducing stress. Notably, similar observations in glioblastoma demonstrate that high xCT levels elevate intracellular oxidative stress and trigger apoptosis, especially under combined stressors such as chemotherapy or glutathione depletion [42]. In ovarian cancer, elevated SLC7A11 was associated with improved sensitivity to paclitaxel therapy and increased OS, potentially due to increased autophagy under chemotherapeutic stress via competing endogenous RNA mode of action (ceRNA) [43]. Taken together, these findings emphasize that while xCT overexpression often supports tumor survival, it can also sensitize cells to cytotoxic agents if oxidative or replicative stress surpasses the cellular compensatory protective capacity. In addition, a work by [42] linked metabolic burden and proliferation. It was shown that mTORC2, a critical regulator of amino acid metabolism, can phosphorylate xCT and thus inhibit its activity [44]. From a technical perspective, we acknowledge that our selected method of antibody‐based quantitation of xCT in living cell models was restricted to cell surface epitope exposure. Notably, the cell surface localization of xCT is subject to regulation and to the cell's metabolic state [10]. While using the PDO platform to validate our findings enables a more personalized approach, analyzing bulk RNA sequencing data often ignores the highly relevant biological intra‐tumoral heterogeneity. This might explain why our analysis, which is based on a public dataset, could not identify a statistically significant correlation between patients' response to chemotherapy and xCT transcript abundance (Fig. S2). Nevertheless, our data underscore the tumor‐ and context‐specific roles of xCT in chemotherapeutic responses.

Increased TFRC promotes cellular iron uptake, which can support DNA synthesis and repair but may also facilitate ferroptosis under conditions of redox dysregulation. We identified that cells with high xCT expression also present TFRC protein upregulation, further suggesting a convergence between iron metabolism and the xCT system. Consequently, manipulating iron homeostasis in tandem with xCT and combined standard chemotherapy treatments appears to be a promising therapeutic strategy [45]. While xCT has been shown to be essential in multiple contexts for sustaining tumor growth [46], our finding that 5‐FU retained (and even enhanced) efficacy in xCT‐high organoids illustrates how metabolic pathways can exert dual‐edged effects in CRC. These observations align with emerging data from other tumor types, indicating that SLC7A11 can either promote or hinder survival depending on specific cellular conditions [47]. Our data on modulated gene expression, associated with loss of function xCT mutations in colon cancer patients, revealed an interesting perspective on xCT's possible involvement in regulating cancer‐to‐nerve interaction. SGK2, as downregulated in mutated samples, is known to play a pivotal role in neuronal survival and excitability by regulating potassium channels [48]. PRDM8, upregulated in mutant cases, is found to be a central regulator of neuronal development [49]. With the emergence of the neuron‐to‐tumor connection as a potent driver of cancer progression [50], our results hint at a possible promising outlook for further investigation of SGK2 and PRDM8 to regulate chemotherapy resistance in colon cancer and potentially regulate the tumor's connectivity with the neuronal micro‐and macroenvironment. We acknowledge that the scope of our study is limited and that the current data are purely correlative, linking xCT protein levels to therapy response. Additional experiments will therefore be required to clarify whether xCT itself—or its interaction partners—actively modulate these effects. Despite this limitation, we observed clear and reproducible correlation patterns across all treatment conditions, which strengthens our motivation to dissect the mechanisms behind these relationships.

We are aware of several limitations of this study. The lack of a detectable difference in xCT cell surface abundance between normal and tumor PDOs suggests that our organoid model still harbors culture‐related biases that we could not eliminate in this study. Although in a recent study SLC7A11 is shown consistently overexpressed in tumor vs. healthy tissue [51] and shows a strong positive mRNA‐to‐protein correlation *in vivo*, organoid‐specific post‐transcriptional mechanisms may equalize xCT protein levels in both PDO types. A likely explanation is that our culture conditions impose cellular stress, triggering transcriptional upregulation and post‐transcriptional stabilization of xCT in normal and tumor organoids alike [52].

Another limitation of this study is the absence of a third reference dataset comprising sequencing data from healthy, tumor‐free patients. Inclusion of such a dataset would have helped to avoid potential bias arising from tumor‐related metabolic effects on the adjacent normal tissue used in this study. Although we sought to minimize this risk by maximizing the resection distance and reducing possible tumor contamination, the possibility of field cancerization effects cannot be fully excluded and should be taken into consideration.

Of note, as we observed strong and significant trends in the functional PDO analysis, results must be interpreted with caution and may not be not entirely generalizable since we analyzed only a handful of PDO models. We acknowledge that future studies with larger sample sizes, depicting ethnic origin, age, and gender differences in a more comprehensive fashion than we could conduct for this project, as well as performing confirmatory quantification of CD44/TFRC to xCT protein‐level effects *in vitro* such as western blot—with and without drug exposure—are necessary to increase the robustness of our hypothesis.

## 5 Conclusion

In summary, even though we did not observe differential SLC7A11 expression in PDOs, we did find that higher xCT levels correlate with improved chemotherapy response. Coupled with the well‐documented overexpression of xCT in patient tumors, these data support xCT as a predictive biomarker. We hypothesize that CRC patients prone to receive neoadjuvant/adjuvant chemotherapy may benefit from pre‐treatment screenings to quantify xCT activation and subsequent stratification into high and low responsiveness to standard chemotherapy treatment. We highlight the possibility of improving standard of care chemotherapy when combining therapy with xCT inhibitor treatment in tumors with high xCT expression. While the role of xCT in CRC and other cancer entities had been investigated before, we were able to newly show the predictive capacity of xCT for 5′ FU therapy sensitivity. By using patient‐derived organoids as more advanced disease models, compared to the variety of 2D cell culture studies up to now, our findings support and add strong evidence of xCT as a promising diagnostic and therapeutic target in colon cancer. Lastly, our data indicate xCT may be a regulator involved in transducing cancer cell interaction with the neuronal microenvironment of colon cancer. Follow‐up studies to decipher the roles of xCT and correlated genes for tumor connectivity are warranted.

## Conflict of interest

The authors declare no conflict of interest.

## Author contributions

MS, KZ, MB, TW, KH, OK, ML, UDK: Conceptualization, data curation, formal analysis; MS, KZ, OK, ML, UDK: writing original draft and review draft; HF, MD, MA, WS, BJ, FB, DS, DM: Reviewing and Advisory. RC, ML, UDK: Funding acquisition, methodology, project administration, resources, supervision. All authors contributed to the article and approved the submitted version.

## Supporting information


**Data S1.** Supporting Information.

**Text S1.** List of reagents used for organoid culture.

**Fig. S1.** Expression levels of SLC7A11 PPI core set.
**Fig. S2.** Expression levels of genes of the Xc^−^ system in relation to drug treatments.
**Fig. S3.** Representative flow cytometric gating strategy.

**Table S1.** Number of Healthy and tumor samples per patient.

**Table S2.** Clinical parameters of the CRC cohort used for the transcriptomic study.

**Table S3.** DEGs from RNA‐seq results.

**Table S4.** Results for hallmarks (MSigDB H).

**Table S5.** Tissue colon cell types. Extracted from HPA for 12 colon cell types.

**Table S6.** Venn diagram of the 100 top codependent CRISPR‐Cas9 from DEPMAP.

**Table S7.** MuTarget results for COAD for target mode.
**Table S8.** DEMs of all miRNA based on sample and DE for miRNA‐seq analysis.

## Data Availability

The data discussed in this publication have been deposited in NCBI's Gene Expression Omnibus (Edgar et al., 2002) and are accessible through GEO Series accession number GSE305337 (https://www.ncbi.nlm.nih.gov/geo/query/acc.cgi?acc=GSE305337).
