# Supplementary material for: Patient‐specific pharmacogenomics demonstrates xCT as predictive therapeutic target in colon cancer with possible implications in tumor connectivity
Source: Mol Oncol. 2025 Sep 24;20(2):428–46. doi: 10.1002/1878-0261.70129 (PMC12936422; doi:10.1002/1878-0261.70129)
Supplement: Supplementary file 3 — Fig. S1. Expression levels of SLC7A11 PPI core set. Fig. S2. Expression levels of genes of the Xc− system in relation to drug treatments. Fig. S3. Representative flow cytometric gating strategy. [file MOL2-20-428-s008.docx]

**Supplementary Figures**


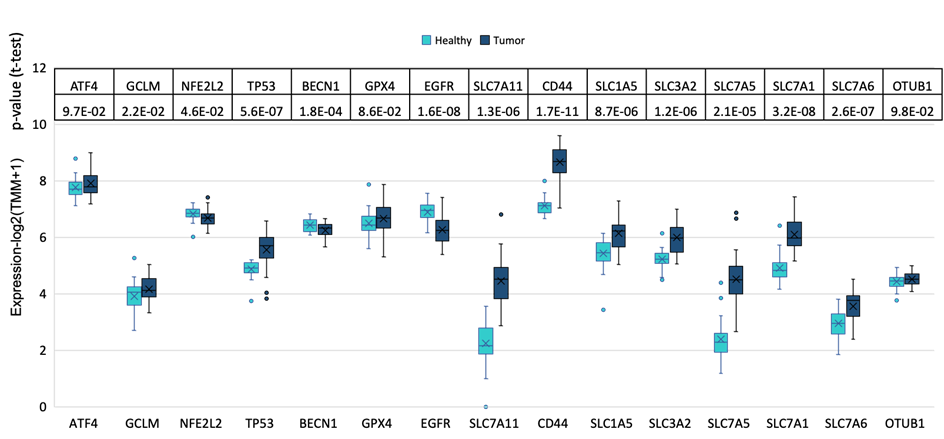


***Figure S1.*** *Box plots of expression on all 32 patients with healthy (light blue) and tumor (dark blue) samples of the associated proteins based on STRING PPI (SLC7A11 core-set). There are 16 core-set, only SLC1A2 is not shown due to low expression level. The statistical p-values by t-test are shown for each gene.*


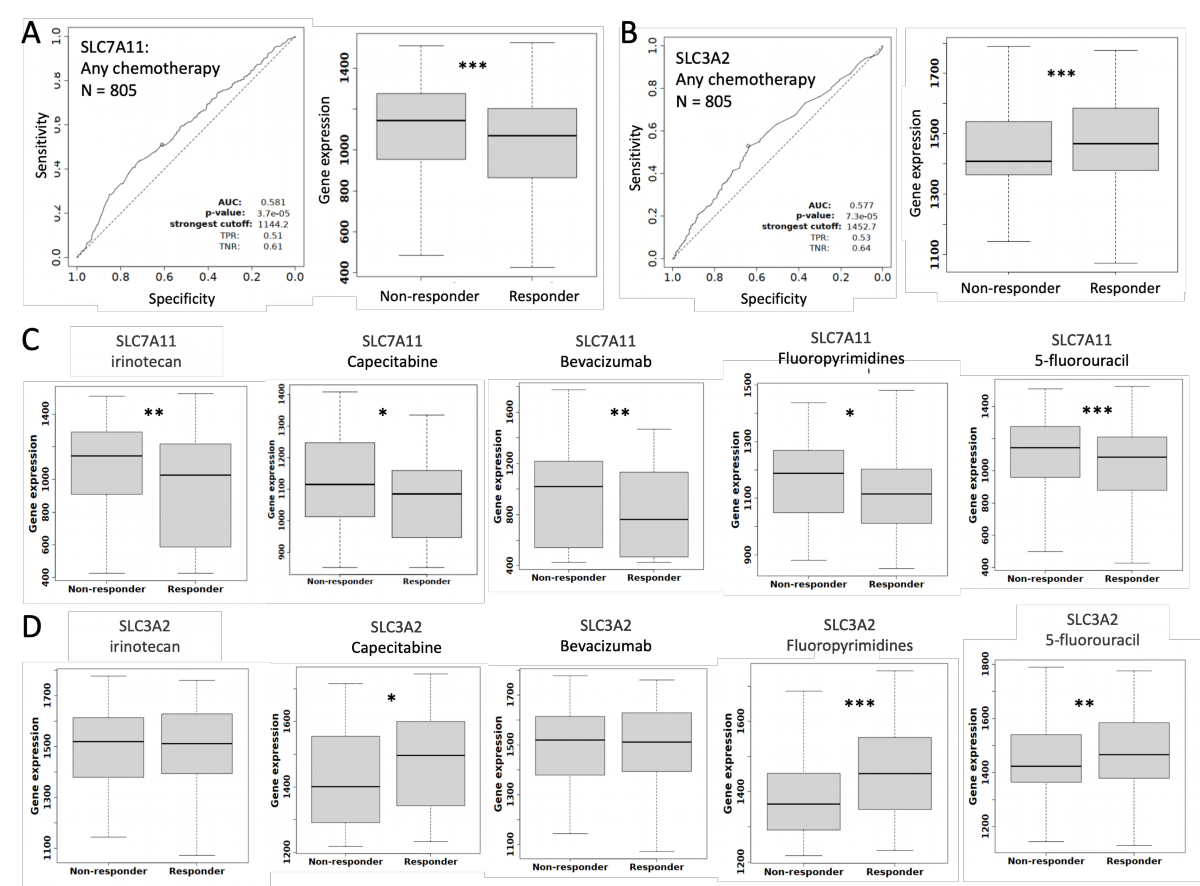


**Figure S2.** Expression levels of genes of the Xc⁻ system in relation to drug treatments and patient responsiveness were analyzed using the KM-Plotter portal (KM-ROC). RNA sequencing was performed on 805 COAD patients, subdivided into responders (n = 451) and non-responders (n = 354) according to RECIST criteria. (**A**) For all chemotherapy treatments, ROC-AUC values and p-values are reported for SLC7A11. (**B**) ROC-AUC values and p-values are also reported for SLC3A2 across all chemotherapy treatments. (**C**) Box plot analyses illustrate the expression levels of SLC7A11 in responders and non-responders for specific agents used in CRC treatment. (**D**) Similarly, box plot analyses show the expression levels of SLC3A2 in responders and non-responders for these agents. Statistical significance is indicated by p-values as follows: <0.05 (*), <0.005 (**) to <0.0005 (***) Results for treatments applied to fewer than 100 patients are not shown.


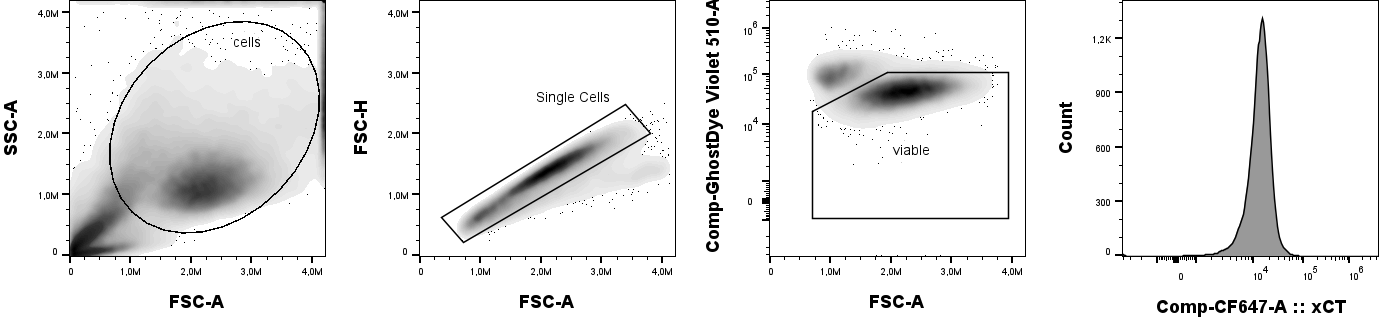


B

A


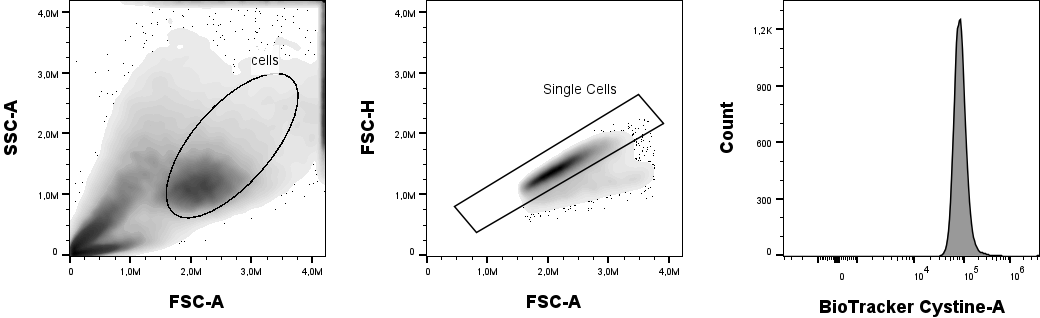


C


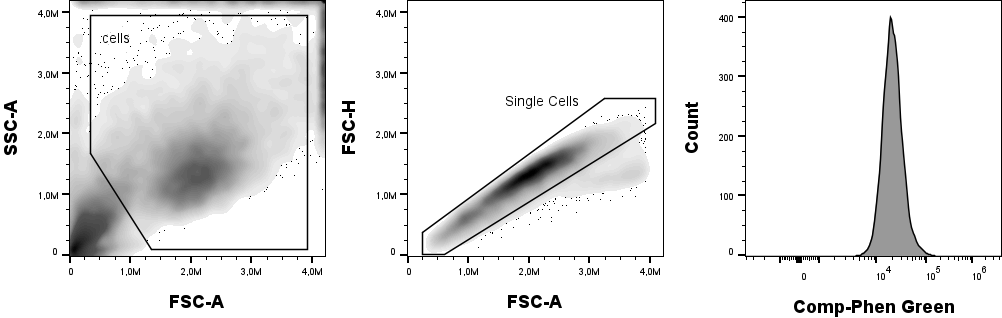


**Figure S3.** Representative flow-cytometric gating strategy for the quantification of cell-surface x-CT, intracellular cystine and the labile iron pool in T86 PDOs exemplary for all PDOs measured. Forward scatter (FSC-A) versus side scatter (SSC-A) density plots were first used to identify the main cell population, after which doublets were excluded by FSC-H versus FSC-A gating and viable singlets were selected through Ghost-Dye Violet 510 exclusion. Panel **A** shows the resulting histogram of CF647 fluorescence corresponding to antibody-labelled x-CT (SLC7A11) on the plasma membrane. Using the identical hierarchical gates, panel **B** presents BioTracker Cystine fluorescence as a read-out of intracellular cystine content, whereas panel **C** displays Phen Green fluorescence, which decreases upon chelation of ferrous and ferric ions and therefore inversely reflects the size of the free iron pool. Histograms are representative of three independent experiments in which at least 10 000 viable singlets were acquired per sample; the percentages annotated in the dot plots indicate the fraction of events retained from the preceding gate
